# Supplementary figures and images for: A low pre-existing anti-NS1 humoral immunity to DENV is associated with microcephaly development after gestational ZIKV exposure
Source: PLoS Negl Trop Dis. 2025 Jan 6;19(1):e0012193. doi: 10.1371/journal.pntd.0012193 (PMC11723597; doi:10.1371/journal.pntd.0012193)

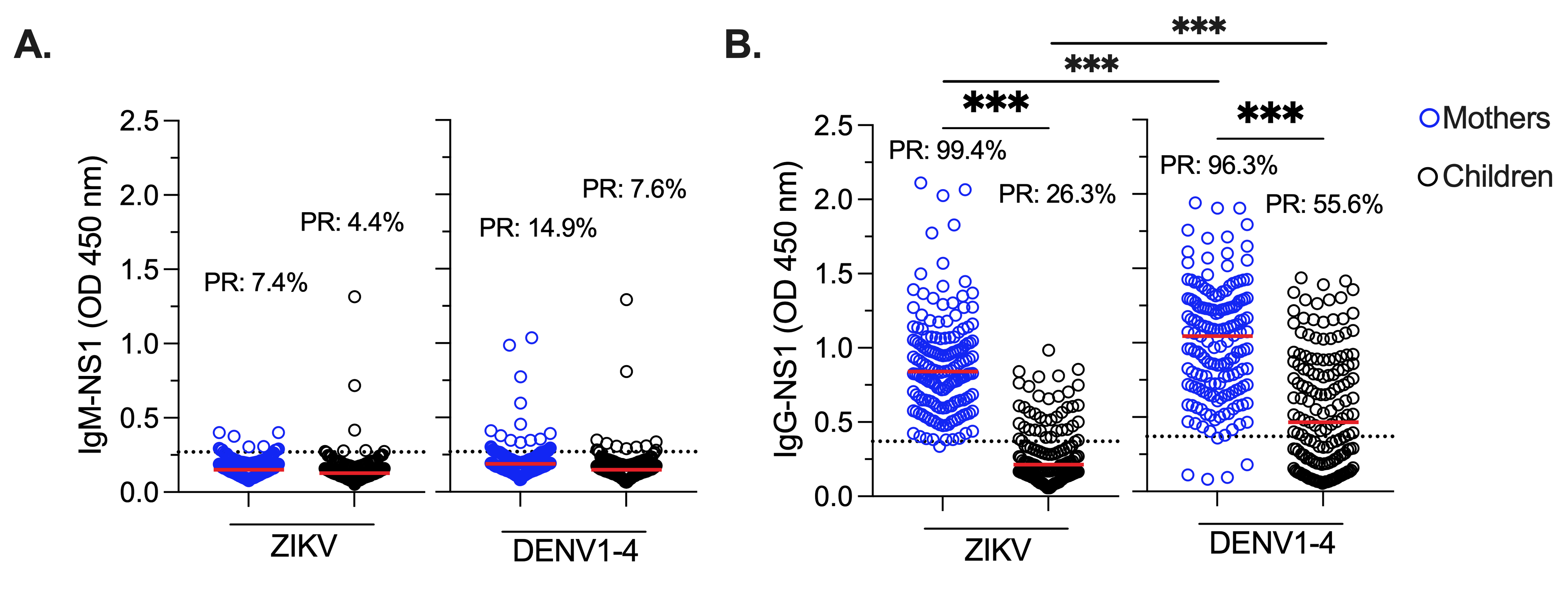

Supplement: S1 Fig — In all the cases, the positivity rate (PR) and the p-value of the Mann-Whitney test are shown. The dashed lines indicate the ELISA limit of detection. ***p < 0.001. (TIF) [file pntd.0012193.s001.tif]

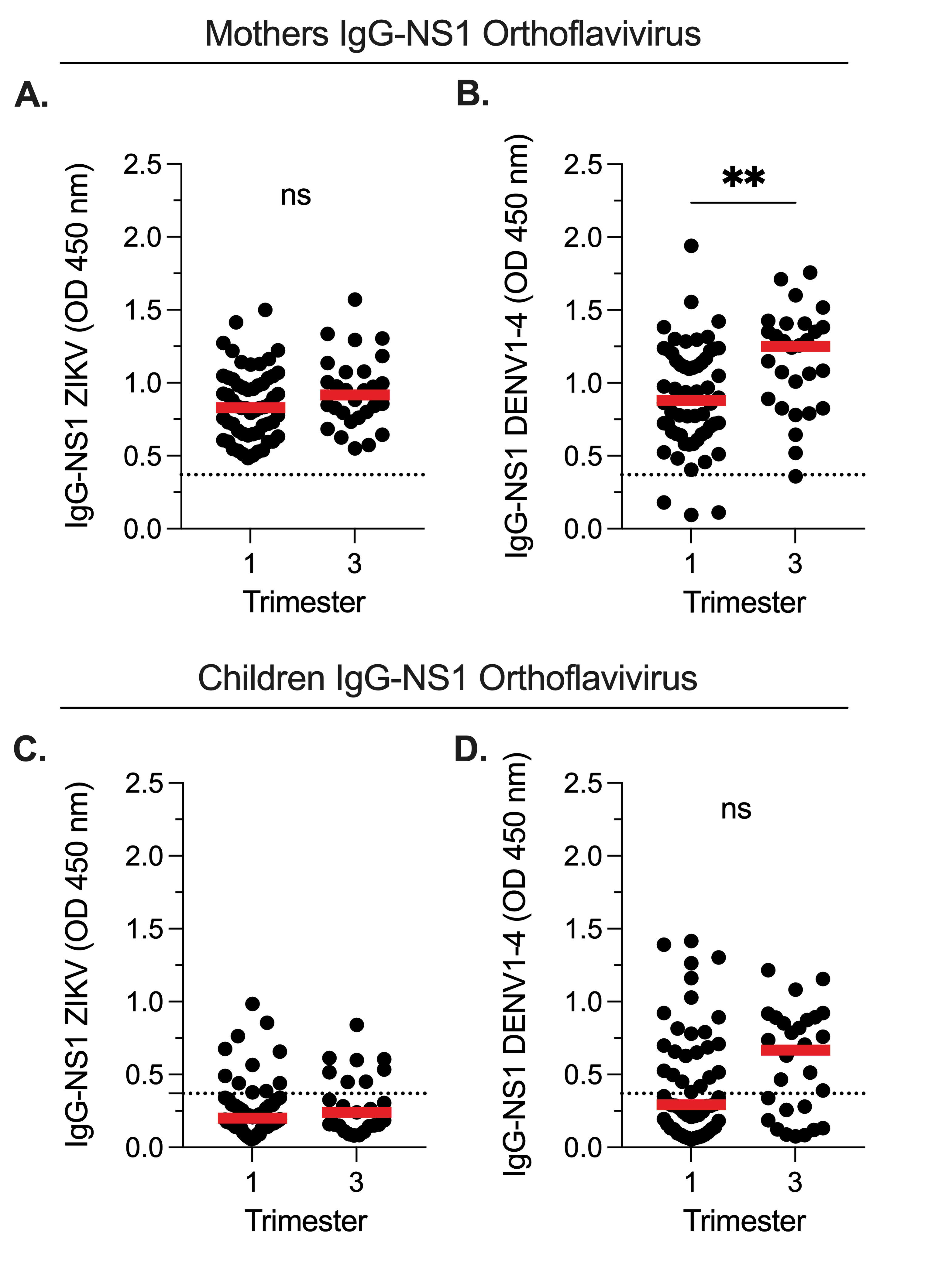

Supplement: S2 Fig — In all the cases, the p-value of the Mann-Whitney test is shown. The dashed lines indicate the ELISA limit of detection. NS: Not statistically significant. **p < 0.01. (TIF) [file pntd.0012193.s002.tif]

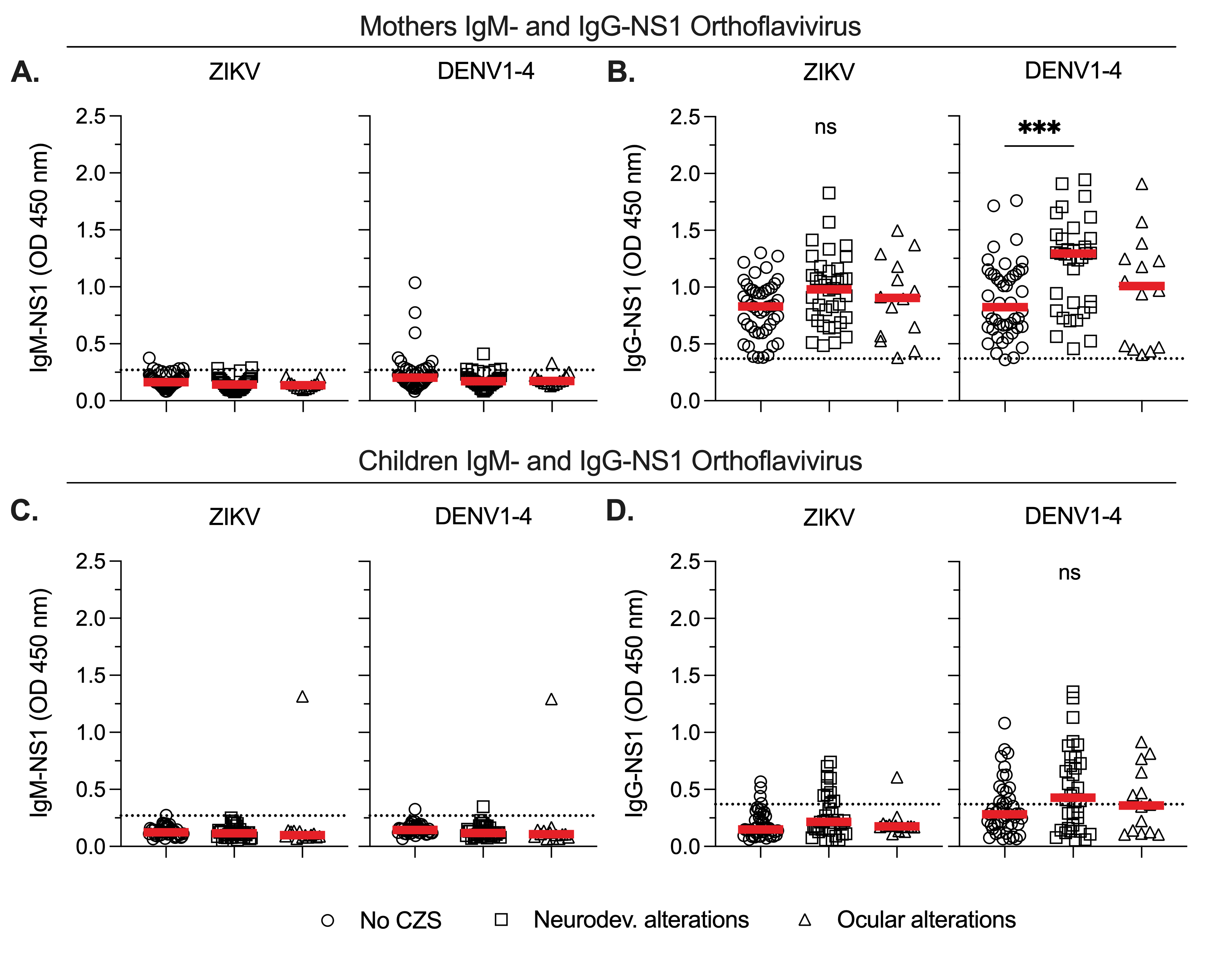

Supplement: S3 Fig — The p-value of Dunn’s test is shown. The dashed lines indicate the ELISA limit of detection. NS: Not statistically significant. ***p < 0.001. (TIF) [file pntd.0012193.s003.tif]
