# Supplementary material for: A low pre-existing anti-NS1 humoral immunity to DENV is associated with microcephaly development after gestational ZIKV exposure
Source: PLoS Negl Trop Dis. 2025 Jan 6;19(1):e0012193. doi: 10.1371/journal.pntd.0012193 (PMC11723597; doi:10.1371/journal.pntd.0012193)
Supplement: S1 Table — (DOCX) [file pntd.0012193.s004.docx]

**SUPPLEMENTAL TABLE**

**Supplementary Table 1. Gestational age of symptoms onset in mothers of children without CZS and with mild CZS (ocular and neurodevelopmental alteration).**

| **Gestational age of symptoms onset; n (%)** | **Study groups** | | **p-value^a^** |
| --- | --- | --- | --- |
|  | **No-CZS  (n=46)** | **Mild-CZS**  **(n=50)** |  |
| Trimester 1 (0-13 weeks) | 27 (58) | 17 (35) | 0.02 |
| Trimester 2 (14-26 weeks) | 11 (25) | 22 (44) | 0.05 |
| Trimester 3 (>26 weeks) | 8 (17) | 10 (21) | 0.79 |

^a^Fisher’s exact test.
